# Development of the ParaOesophageal hernia SympTom (POST) tool

**DOI:** 10.1093/bjs/znac139

**Published:** 2022-05-31

**Authors:** Aiysha Puri, Nikhil M Patel, Viknesh Sounderajah, Lorenzo Ferri, Ewen A Griffiths, Donald Low, Nick Maynard, Carmen Mueller, Manuel Pera, Mark I van Berge Henegouwen, David I Watson, Giovanni Zaninotto, George B Hanna, Sheraz R Markar, the POST Collaborative, R Aye, B Louie, R Baigrie, L Bonavina, G Darling, P M Fisichella, S Jaume-Bottcher, J C Lipham, W S Melvin, K Nason, B Oelschlager, F Puccetti, R Rosati, J S Roth, P Siersma, B Smithers, N Soper, S Thompson

**Affiliations:** Department of Surgery and Cancer, Imperial College London, London, UK; Department of Surgery and Cancer, Imperial College London, London, UK; Department of Surgery and Cancer, Imperial College London, London, UK; Division of Thoracic and Upper Gastrointestinal Surgery, McGill University Health Centre, Montreal, Quebec, Canada; Department of Upper Gastrointestinal Surgery, Queen Elizabeth Hospital Birmingham, University Hospitals Birmingham NHS Foundation Trust, Edgbaston, Birmingham, UK; Institute of Cancer and Genomic Sciences, College of Medical and Dental Sciences, University of Birmingham, Birmingham, UK; Department of Thoracic Surgery and Thoracic Oncology, Virginia Mason Medical Centre, Seattle, Washington, USA; Oxford Oesophagogastric Centre, Churchill Hospital, Oxford University Hospitals NHS Foundation Trust, Oxford, UK; Division of Thoracic and Upper Gastrointestinal Surgery, McGill University Health Centre, Montreal, Quebec, Canada; Department of Surgery, University Hospital del Mar, Barcelona, Spain; Hospital del Mar Medical Research Institute (IMIM), Barcelona, Spain; Department of Surgery, Amsterdam University Medical Centres, Cancer Centre Amsterdam, Amsterdam, the Netherlands; Flinders University, Discipline of Surgery, Flinders Medical Centre, Adelaide, South Australia, Australia; College of Medicine and Public Health, Flinders University, Adelaide, South Australia, Australia; Department of Surgery and Cancer, Imperial College London, London, UK; Department of Surgery and Cancer, Imperial College London, London, UK; Department of Surgery and Cancer, Imperial College London, London, UK; Department of Molecular Medicine and Surgery, Karolinska Institutet, Stockholm, Sweden; Nuffield Department of Surgery, University of Oxford, Oxford, UK

## Abstract

**Background:**

The aim of this study was to develop a symptom severity instrument (ParaOesophageal hernia SympTom (POST) tool) specific to para-oesophageal hernia (POH).

**Methods:**

The POST tool was developed in four stages. The first was establishment of a Steering Committee. In the second stage, items were generated through a systematic review and online scoping survey of international experts. In the third stage, a three-round modified Delphi consensus process was conducted with a group of international experts who were asked to rate the importance of candidate items. An *a priori* threshold for inclusion was set at 80 per cent. The modified Delphi process culminated in a consensus meeting to develop the first iteration of the tool. In the final stage, two international patient workshops were held to assess the content validity and acceptability of the POST tool.

**Results:**

The systematic review and scoping survey generated 64 symptoms, refined to 20 for inclusion in the modified Delphi consensus process. Twenty-six global experts participated in the Delphi consensus process. Five symptoms reached consensus across two rounds: difficulty getting solid foods down, chest pain after meals, difficulty getting liquids down, shortness of breath only after meals, and an early feeling of fullness after eating. The subsequent patient workshops deemed these five symptoms to be relevant and suggested that reflux should be included; these were taken forward to create the final POST tool.

**Conclusion:**

The POST tool is the first instrument designed to capture POH-specific symptoms. It will allow clinicians to standardize reporting of symptoms of POH and evaluate the response to surgical intervention.

## Teaser

In order to generate a symptom severity instrument specific to para-oesophageal hernia (POH), a three round modified Delphi consensus study of expert clinicians was undertaken. Five symptoms met the prespecified threshold for inclusion in the Para-Oesophageal hernia SympTom (POST) tool; all of which were obstructive in nature. The content validity and acceptability of the first iteration tool was examined through patient design workshops, which prompted the addition of a six symptom, reflux, to the now finalized tool.

## Introduction

Patients with para-oesophageal hernia (POH) often report a broad range of symptoms that can either individually or cumulatively have a substantial impact on their quality of life^1^. These symptoms are not only gastrointestinal in nature but can also affect respiratory and cardiovascular systems^2–4^. Symptom severity tools are often used in benign upper gastrointestinal surgery to determine the severity of symptoms and to assess the benefit of surgical intervention. However, at present, there is no symptom severity tool that is specific to POH, and the broad range of symptoms associated with this pathology. As such, there remains no uniform means of either assessing eligibility for, or defining success after, surgery. It is well understood that the incidence of POH increases with age; however, the older patient typically has additional co-morbidities, reduced physiological fitness, and frailty. These conditions are associated with increased morbidity and mortality when undergoing surgical intervention^5^. Thus, the decision to offer surgery can be challenging in this cohort of patients. A dedicated symptom severity tool could help evaluate thresholds for surgical intervention for POH and the symptomatic response to surgical intervention, in both clinical trials and applied clinical practice settings.

To address this need, the ParaOesophageal hernia SympTom (POST) tool Collaborative aimed to develop a specific symptom tool for the clinical assessment of POH by means of a multistage evidence generation process. The first part of this work was to undertake a systematic review of the published literature to identify the range of symptoms and health-related quality of life (HRQoL) and symptom severity tools used previously to assess POH^6^. This systematic review revealed that there is a broad range of symptoms associated with POH, and that a symptom severity tool that encompasses them adequately is lacking. Currently, the most commonly used symptom severity tools used to assess POH focus on gastro-oesophageal reflux disease (GORD)^6^, likely driven by the fact that the observed incidence of GORD in patients with POH or hiatal hernias is as high as 86 per cent^7^. However, the main drivers of surgical intervention in POH (especially types II–IV) are often other non-gastrointestinal symptoms that may not be evaluated routinely in patients as they are not accounted for by these tools.

The aim of this study was to develop a symptom severity instrument (POST tool) specific to POH.

## Methods

This study was conducted in accordance with guidance from both the COnsensus-based Standards for the selection of health Measurement INstruments (COSMIN) initiative and Core Outcome Measures in Effectiveness Trials (COMET) initiative^8^. As such, the development of the POST tool can be distilled into four stages.

### Stage 1: project organization

Study management was conducted by a Project Team and a wider Steering Committee. The Project Team (4 authors) was responsible for identifying suitable members of the Steering Committee, initial evidence generation, undertaking the modified Delphi consensus process, organizing the consensus meeting, and drafting material related to the POST Collaborative. In addition to the Project Team, a wider Steering Committee, consisting of 10 clinicians from Europe, North America, and Australasia, was established to provide specialist guidance throughout. These individuals were identified through their established clinical expertise in the field of POH repair.

### Stage 2: candidate item generation

Candidate items for the POST tool were derived from the study group’s preceding published systematic review as well as an online scoping survey of the shortlisted panel of experts using specific surveying software, Google Forms (Google, USA). Leading surgeons and gastroenterologists from around the world who regularly manage patients with POH were invited to participate in this process. These participants were identified as expert POH clinicians, being lead authors from the highest-volume series identified by the systematic review, or based on their established clinical expertise as representatives from relevant societal bodies, including the International Society for Diseases of Esophagus, European Society for Disease of Esophagus, European Association of Endoscopic Surgery, United European Gastroenterology, Society for Surgery of the Alimentary Tract, and Society for American Gastrointestinal Endoscopic Surgeons^6^. The scoping survey allowed the capture of items that may not necessarily have been addressed or highlighted by the systematic review, which served as an additional means of rigour. Moreover, the scoping survey captured current practice around symptom assessment tools, modes of investigation, operative thresholds, and surveillance strategies among respondents.

### Stage 3: modified Delphi consensus process

The Delphi consensus methodology is a well established method of obtaining a collective opinion from a group of experts through a series of iterative questionnaires; each of these is refined based on feedback from respondents to a previous version^9^. The specific goal of this Delphi consensus process was to generate the first iteration of the POST tool over three rounds. The first two rounds were conducted through Qualtrics XM. On account of the ongoing COVID-19 pandemic, an in-person meeting (third round) was deemed infeasible and the meeting was therefore conducted virtually through Zoom (Zoom Video Communications, San Jose, California, USA). The choice of experts for this process mirrored that in stage 2.

#### Round 1

Round 1 of the modified Delphi consensus process comprised a questionnaire formulated by the Project Team. This questionnaire included a list of symptoms associated with POH yielded from stage 2. Participants were asked to rate the level of importance of each symptom in assessing a patient with POH by using a five-point Likert scale; a score of 1 indicated that a symptom was unimportant, and 5 meant that a symptom was critically important in the assessment of such a patient. The threshold for inclusion was set at 80 per cent or higher. Symptoms that reached a rating of at least 80 per cent score 4 or 5 were deemed to be essential for inclusion and were discussed in the consensus meeting. Symptoms that did not reach this threshold for inclusion were put forward for the next round of the modified Delphi consensus. Symptoms that reached a rating of at least 80 per cent score 1 or 2 were excluded from the process. Moreover, participants were asked in free-text format to suggest symptoms that they considered to be important in assessing patients with POH. The results of round 1 were anonymized and analysed to determine the level of consensus on each symptom. The results were presented to all participants in the form of bar charts to illustrate the level of consensus on each symptom before participation in round 2.

Furthermore, based on the results of round 1, it was determined that some symptoms would be better assessed before surgery and some would be more appropriately be assessed after operation; this was also taken into consideration when setting up round 2.

#### Round 2

Round 2 followed a near-identical structure to round 1 in that each symptom was scored using a five-point Likert scale; however, items were also assessed regarding their importance before and after operation. Participants were again invited to submit any additional symptoms they felt to be important in perioperative assessment of patients with POH. The participants from round 1 were invited by e-mail to take part in round 2. The results of round 2 were collated and analysed before being discussed by the members of the POST Collaborative in a further video conference.

#### Round 3

Attendees of the Zoom meeting were members of the Steering Committee. The primary objective was to develop a draft version of the POST tool. As recommended in the COMET handbook^10^, the nominal group technique, a highly structured group interaction framework, was used to aid this process. Following a brief introduction and explanation of the purpose of the meeting by the facilitators, participants discussed the inclusion and exclusion of candidate items. This discussion phase was led by the facilitators to ensure that the discussion was not dominated by any one individual and was as neutral as possible^11^.

### Stage 4: patient design workshops

Following the development of the tool, patient workshops were held in the UK and Spain. Patients who had previously undergone repair of POH were identified by surgeons in the POST Collaborative for inclusion in the workshops. The patient design workshops were undertaken using Microsoft^®^ Teams (Microsoft, Redmond, Washington, USA). The objective of these discussions was two-fold: to further identify issues not uncovered during previous evidence generation steps; and to gain further understanding of the perceived importance of specific items that had already been raised. An expert, native language-speaking facilitator led the discussion at both workshops. Anonymized discussion transcripts were maintained, which allowed thematic analysis of content. New themes and items that were not identified as part of the previous evidence generation process were presented back to the Project Team and Steering Committee for further discussion.

## Results

### Scoping survey

There were 25 respondents to the scoping survey (*Table 1*), of whom eight reported carrying out more than 50 POH repairs per year and four who performed 10 or fewer POH repairs per year. The majority were from North America (52 per cent).

**Fig. 1:**
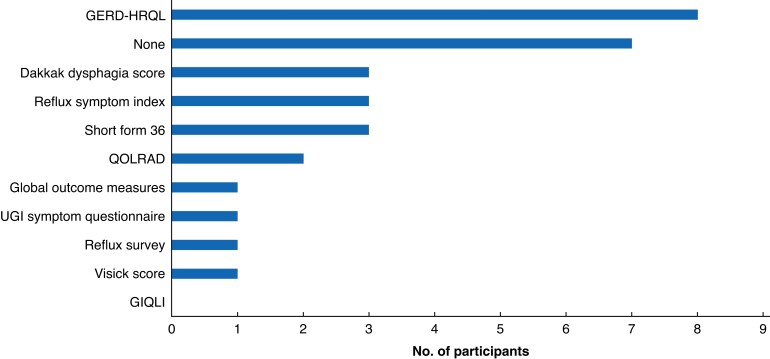
Routine use of health-related quality-of-life and symptom severity tools by participants of scoping survey GERD-HRQL, Gastro-oesophageal Reflux Disease Health Related Quality of Life; QOLRAD, Quality of Life in Reflux and Dyspepsia Questionnaire; UGI, upper gastrointestinal; GIQLI, Gastrointestinal Quality of Life Score.

Routine preoperative assessment consisted of oesophagogastroduodenoscopy and contrast swallow (*Fig. S1*). Clinicians typically used either the Gastro-oesophageal Reflux Disease Health Related Quality of Life (GERD-HRQL) tool or no quality-of-life tool for preoperative assessment (*Fig. 1*). The majority of respondents (96 per cent) screened for gastrointestinal symptoms as part of the preoperative evaluation, including heartburn/GORD/reflux, and dysphagia/odynophagia. Dyspnoea was considered by 18 of 25 clinicians in the preoperative work-up. Most respondents rated symptoms and clinical assessment as a 4 or 5 of 5 on the Likert scale for importance when deciding to offer operative treatment for POH. The systematic review and scoping survey generated 64 symptoms.

Twenty items derived from the systematic review and scoping survey were taken through to the modified Delphi consensus process. These were categorized in three symptom categories: gastrointestinal, respiratory, and other.

### Modified Delphi consensus process

The first round was conducted from 4 to 25 February 2021, the second round from 17 March to 9 April 2021, and the third round on 15 June 2021.

### Round 1

A total of 26 responses were received in round 1 (25 surgeons, 1 gastroenterologist).

**Fig. 2:**
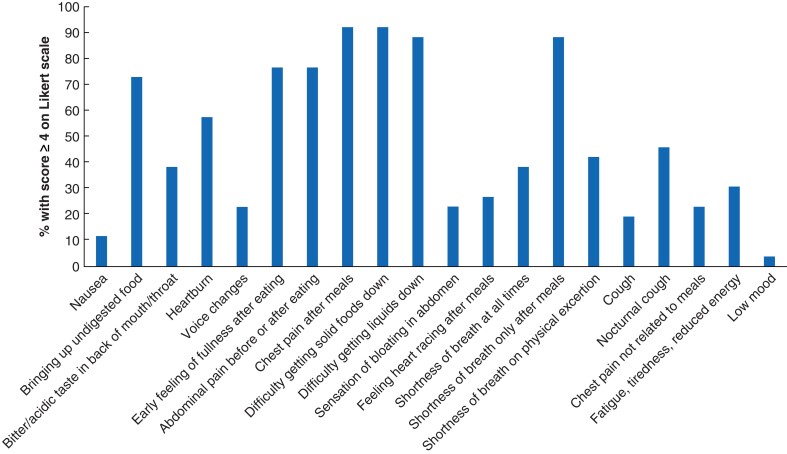
Modified Delphi round 1 results

Three symptoms in the gastrointestinal category met the prespecified consensus threshold (above 80 per cent) for inclusion in the POST tool, subject to group discussion in round 3. These were chest pain after meals (92 per cent consensus), difficulty getting solid foods down (92 per cent consensus), and difficulty getting liquids down (88 per cent consensus) (*Fig. 2*). Four symptoms in the gastrointestinal category reached between 40 and 79 per cent consensus, and these were re-evaluated in round 2: early feeling of fullness after eating (77 per cent consensus), abdominal pain before or after eating (77 per cent consensus), bringing up undigested food (73 per cent consensus), and heartburn (58 per cent consensus). Only shortness of breath after meals (88 per cent consensus) reached above 80 per cent consensus in the respiratory category, whereas two symptoms reached between 40 and 79 per cent consensus and were revaluated in round 2: shortness of breath on physical exertion (42 per cent consensus) and nocturnal cough (46 per cent consensus). No symptoms in the other category reached above 40 per cent consensus. No new items for inclusion in round 2 were generated in the free-text component of round 1. On analysis of round 1 results among the Project Team and Steering Committee, it was highlighted that, in the overall evaluation of patients with POH, the symptoms that had reached consensus were all those useful in evaluating patients before surgery; only some symptoms were relevant after operation. Therefore, it was decided to re-rate all of the symptoms with particular respect to a postoperative context in round 2.

### Round 2

**Fig. 3:**
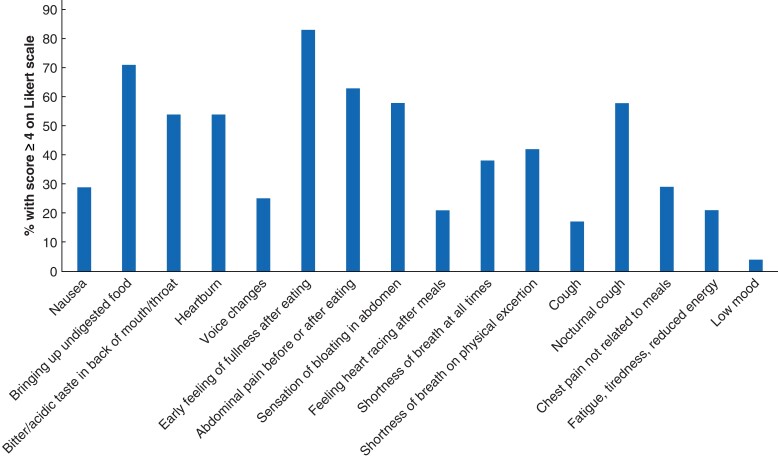
Modified Delphi round 2 results

Twenty-four of 26 respondents from the scoping round responded to round 2. Of all the items in this round, only early feeling of fullness after eating reached consensus (83 per cent) (*Fig. 3*). When considering inclusion of specific items for a postoperative context, none of the symptoms reached consensus for inclusion and they were not considered further.

### Round 3

The consensus meeting was attended by all 14 members of the Project Team and Steering Committee. At the consensus meeting, a preliminary tool was created and presented to the group, containing the five symptoms that had reached above 80 per cent consensus across both previous rounds. Moreover, the precise scope of the tool was agreed on; the POST tool was intended to be patient-facing and should incorporate self-reported frequency of symptoms and self-reported impact on quality of life, in addition to the identification of problem symptoms.

### Patient workshop

In the UK and Spain, design workshop participants were recruited by members of the Steering Committee. Participants were identified through their involvement in charitable organizations which raise awareness for those who have undergone POH repair (*Appendix S1*). Each design workshop participant had previously undergone POH surgery. All indicated that the tool was readable, understandable, and easy to complete. The majority (88 per cent) felt that the symptoms captured by the modified Delphi process were the primary symptoms they had experienced before operation. Patients felt that the tool could be used in both the preoperative and postoperative settings. All the patients felt that reflux or heartburn had been a significant component of their symptoms, and should be included in the tool (*Table 1*). It was decided unanimously among the Project Team and Steering Committee that reflux warranted inclusion in the POST tool on the basis of this consistent feedback.

**Table 1:** Final POST tool

Symptom	Consensus (%)	Do you have any of the following symptoms and how often? Please mark				How much impact does the symptom have on your life? Please mark (0 = none, 5 = severe)					
		Never 0	Monthly 1	Weekly 2	Daily/multiple times per day 3						
**1. Difficulty getting solid foods down**	92 (round 1)	◯	◯	◯	◯	0	1	2	3	4	5
**2. Difficulty getting liquids down**	88 (round 1)	◯	◯	◯	◯	0	1	2	3	4	5
**3. Chest pain after meals**	92 (round 1)	◯	◯	◯	◯	0	1	2	3	4	5
**4. Shortness of breath after meals**	88 (round 1)	◯	◯	◯	◯	0	1	2	3	4	5
**5. Early feeling of fullness after eating**	83 (round 2)	◯	◯	◯	◯	0	1	2	3	4	5
**6. Heartburn or reflux**	n.a.	◯	◯	◯	◯	0	1	2	3	4	5

n.a., not applicable.

## Discussion

Using a multiphase evidence generation process, a core set of symptoms that affect patients with POH was identified. These symptoms are not routinely captured by the currently established HRQoL or symptom severity tools used to assess patients with POH. The core symptom set has been concurrently developed into a patient-facing tool that can be used in both preoperative and postoperative settings to assess the symptomatic response to surgical intervention and standardize the reporting of symptoms of POH. This tool was deemed to be acceptable to patients across two international workshops.

Among the panel of experts, the most widely used tool to assess symptoms and quality of life before surgery was the GERD-HRQL. This is consistent with the findings of the study group’s systematic review^6^, in which it was reported that 47.7 per cent of studies used this tool for assessment. Interestingly, comparison of the core symptoms identified by the process described here with symptoms in the GERD-HRQL questionnaire revealed overlap only in the broad term ‘reflux’; the more specific POH symptoms captured in POST do not feature in the GERD-HRQL tool. In addition to reflux, the core symptoms included in POST were: chest pain after meals, difficulty in getting solid foods down, difficulty in getting liquids down, shortness of breath after meals, and early feeling of fullness after eating. Crucially, most of the symptoms included in POST are obstructive-type symptoms rather than regurgitant symptoms. Of note, these symptoms were identified by surgeons and corroborated by patients as the most important in affecting HRQoL, and thus driving surgical intervention.

In developing POST, careful consideration had to be made between developing a completely comprehensive set of symptoms that may be associated with POH and focusing on the core set of symptoms with the greatest impact on HRQoL that could be combined into a pragmatic and clinically useable tool. At the workshops, patients did identify with other symptoms, including low mood and fatigue; however, these were not included in the final version of POST as they were not felt to be specific to POH.

The next step in the development of POST is to further assess its content validity and clinical usability. To do this, the tool will be tested in an observational international cohort study. This will include administration of the tool before and after POH repair (up to 8 months after surgery) to evaluate how the tool performs longitudinally and, furthermore, how it may be integrated into the clinical pathway to identify treatment success.

It is important to consider the strengths and weakness of the Delphi methodology that led to the development of POST. Strengths of the process are that an international panel of expert surgeons were able to reach consensus, and the interest of the group in the project was high as reflected by the low drop-out rate. A weakness of the process was the large representation from Europe and North America; however, this reflects the published literature on POH^6,12–14^. Furthermore, most participants were from high-income countries, and the content validity of this tool in low- and middle-income countries remains unknown. A further potential limitation was that the majority of respondents were surgeons, with only one gastroenterologist included; this reflects the surgical nature of symptom assessment for POH and consideration of surgical success. Despite this, the patients’ perspective was sought in two different countries and taken into consideration in development of the final tool. However, including patients from only two countries means that the external validity of the tool internationally could be questioned. This will be tested in the planned future observational international cohort study. Furthermore, these patients were selected as suitable by surgeons taking part in the Delphi process; however, data were not collected on the operative outcome of these patients, and personal experience may have been an important confounder when patients were asked to discuss the relative importance of symptoms.

In conclusion, the POST collaborative group has developed an evidence- and expertise-based symptom tool to assess patients undergoing POH surgery. This tool includes six symptoms, the majority focused on the obstructive symptoms associated with POH. Future investigations are required to test prospectively the content validity and clinical utility of this POH-specific symptom tool.

## Collaborators

POST Collaborative: R. Aye, B. Louie (Swedish Cancer Institute and Medical Centre, Seattle, USA); R. Baigrie (Kingsbury Hospital and Groote Schuur Hospitals, Cape Town, South Africa); L. Bonavina (University of Milan, Milan, Italy), G. Darling (Toronto General Hospital, Toronto, Canada); P. M. Fisichella (Northwestern University Feinberg School of Medicine, Chicago, USA); S. Jaume-Bottcher (University Hospital del Mar, Barcelona, Spain); J. C. Lipham (University of Southern California, Los Angeles, USA); W. S. Melvin (Montefiore Medical Centre, New York, USA); K. Nason (University of Massacheusetts Chan Medical School, Springfield, USA); B. Oelschlager (University of Washington, Seattle, USA); F. Puccetti, R. Rosati (San Raffaele Hospital, Milan, Italy); J. S. Roth (University of Kentucky, Lexington, USA); P. Siersma (University Medical Centre Utrecht, Utrecht, The Netherlands); B. Smithers (University of Queensland, Woolloongabba, Australia); N. Soper (University of Arizona College of Medicine, Phoenix, USA); S. Thompson (Flinders University, Adelaide, Australia).

## Supplementary Material

znac139_Supplementary_Data
