# Supplementary material for: Development of the ParaOesophageal hernia SympTom (POST) tool
Source: Br J Surg. 2022 May 31;109(8):727–32. doi: 10.1093/bjs/znac139 (PMC10364681; doi:10.1093/bjs/znac139)
Supplement: znac139_Supplementary_Data [file znac139_supplementary_data.zip › Appendix S1.docx]

**Appendix S1: PATIENT WORKSHOPS**

Patient workshops were held in the United Kingdom (UK) and Spain to assess acceptability of POST.

*Methods*

**UK Workshop**

Patients of surgeons within the POST Collaborative (NM and MP) who had previously undergone repair of POH were invited to participate. They were contacted by telephone and were sent information sheets describing the structure and purpose of the POST study and workshop. They were asked to sign and return consent forms to confirm their participation. The workshop in the UK was held on Friday 8^th^ September 2021 and took place online using Microsoft Teams (Microsoft Corporation, USA) for which a link was sent to consenting members. Semi-structured interviews were carried out by members of the POST collaborative (NP, AP, VS). For the duration of the workshops, all participants were asked to keep their web-cameras off to preserve confidentiality.

The semi-structured interviews focused on the following three themes; accuracy of the current version of POST at assessing POH, usability of POST and the inclusion of other symptoms initially excluded from POST as a result of the literature review and Delphi consensus.

**Spanish Workshops**

Two in-person workshops were run at Hospital del Mar, Barcelona, Spain on Wednesday 24^th^ November and Monday 29^th^ November 2021 as part of the Para-Oesophageal hernia SympTom (POST) tool study.

Patients were presented with a summary of the findings of the POST study thus far, including the results of the systematic literature review published earlier this year, and were asked to review the symptom tool in its current format. Furthermore, they were asked to reflect on their own experiences of life with a para-oesophageal hernia and answer the following 4 questions:

1. In your opinion, are the symptoms presented in this tool correct? Are there other symptoms that you experienced that you feel are important and should be included in this tool?
2. In your opinion, is the way used to assess the frequency of symptoms and the degree of their effect on the patient's life adequate?
3. In your opinion, is this tool accurate to be used for both pre- and post- POH repair patients?
4. Is an electronic version of this tool acceptable?

*Results*

**UK workshop**

A thematic analysis approach was employed to interpret patients’ attitudes towards the POST tool. The workshop was audio-recorded and transcribed verbatim. All identifiable characteristics were removed. The transcript was analysed and coded by NP into three themes based on the answers given in the semi-structured interviews:

1. Accuracy of the POST tool in terms of the symptoms included in the tool in assessing patients with POH (*Table S1*).
2. Additional symptoms reported by the participating patients that should be included in the POST tool (*Table S2*).
3. Usability of the POST tool (*Table S3*).

**Spanish Workshops**

A total of 9 patients who had previously undergone repair of para-oesophageal hernia (POH) from 18 months to 5 years before the date of the first workshop were invited to participate.

The majority of the patients (7 out of 9) that participated in this workshop felt that the symptoms presented in the POST tool were correct. The primary symptom experienced was an early feeling of fullness after eating (6 out of 9 patients), followed by difficulty getting liquids down (3 patients), shortness of breath after meals (3 patients), difficulty getting solid foods down (1 patient), and chest pain after meals (1 patient).

Other symptoms experienced by the participants, but not included in the symptom tool, were heartburn (3 out of 9 patients), bringing up indigested food (3 patients), abdominal pain (2 patients), cough (2 patients), sensation of bloating in the abdomen (1 patient), and voice changes (1 patient).

Just over half the cohort (5 out of 9 patients) felt that an electronic version of this tool was acceptable to record symptom severity and impact on quality of life caused by POH.

All 9 patients felt that the way used to assess the frequency of symptoms and the degree of their effect on the patient's life was adequate. All of them also felt that the tool was accurate to be used for both before and after repair of POH.

*Table S4* summarises the answers of this cohort of patients.

*Discussion*

The results of these patient workshops demonstrate overall acceptability of the symptom tool in terms of accuracy of symptoms included and ability to record symptom severity, and impact on QOL caused by POH before repair as well as accurately assess for resolution of symptoms after repair. It was reassuring to find that in a separate cohort of patients, who have all undergone primary surgery for POH with simultaneous anti-reflux procedure, that there is a consistency in the symptoms experienced. This extrinsically validates the findings of the modified Delphi consensus.

After presenting the results of the systematic review and outcomes of the Delphi consensus, the discussion was opened to feedback on the current version of POST while inviting patients to discuss their own symptoms and experiences with POH, as outlined in Supplementary Tables 2 and 4. This enabled the overall accuracy of the symptoms in POST to be gauged. Of note, patients reported ‘chest pain’ as common with their POH, however, according to the tool specifically, ‘chest pain after meals,’ was highlighted by the Delphi consensus. If the former were to be used, this may result in patients with other causes of chest pain, such as ischaemic heart disease to be incorrectly captured by the tool as suspicious for having a POH. Additionally, patients reported, ‘recurrent chest infections,’ and, ‘frequent chest colds,’ to be associated with their POH. However, although this was not highlighted in the Delphi consensus as a significant symptom, several studies identified in the systematic review reported symptoms such as, ‘cough,’ ‘sore throat,’ ‘excess mucus,’ and ‘post-nasal drip,’ as common in POH^1^. Recurrent chest infections may be due to micro aspirations occurring as a result of POH ^2^. This section of the workshop has highlighted that there is a broad range of symptoms caused by POH, which confirms the finding of 82 symptoms reported across 220 studies in the systematic literature review ^1^.

The workshops therefore moved to discussing what additional symptoms the participating patients would like to see included in POST based on their own experience. There was agreement in 50% of patients globally that that ‘reflux,’ should be among the symptoms in the tool. This supported the outcomes of the discussions by the panel of invited experts in the Delphi consensus, and it was reported on 26 occasions by the studies in the systematic review ^1^, that symptoms of POH include reflux. It was therefore deemed that, ‘reflux,’ would be added to POST as an essential symptom in assessing patients with suspected POH and to determine whether repair had been successful. Of note, this section of the workshop highlighted a number of other symptoms such as, ‘shortness of breath at all times,’ ‘bloating,’ and ‘gassiness.’ Of these, only ‘bloating,’ was highlighted in the systematic review by 40 studies ^1^. This supports the notion that there are a wide range of symptoms attributed to POH and suggests that there may be a subjective element to the effects of POH on patients. The patients that participated in this workshop were not asked to declare any of their co-morbidities and therefore there may be an element of overlap between the symptoms caused by underlying health conditions such as asthma which includes ‘shortness of breath,’ and POH. It may therefore be difficult to distinguish between the cause of the patient’s shortness of breath when determining the impact of POH on QOL.

Finally, a discussion on how POST could be improved was held to conclude the workshop. As highlighted in Supplementary Table 4, comments varied from a larger Likert scale to enable a more precise quantification of impact of symptoms on QOL, to including many more symptoms in POST.

Comments on how best to deliver the tool confirmed that both electronic and paper format were required to allow all eligible patients to take part in the study without any potential barriers to participation caused by potential difficulties with technology, and any challenges patients may face with using technology such as electronic forms. Development of an electronic version of the tool to enable easier capture of data was suggested by the POST collaborative and this was supported by the participants of the UK workshop. However, this did not appear to be supported by the cohort in Spain. There may be several reasons for this, such as lack of use of technology in healthcare locally or reduced patient access to or lack of confidence with technology, making these patients less likely to engage with an electronic version of the symptom tool.

One patient felt including all 15 symptoms removed by the Delphi consensus would be appropriate. Of note, this patient reported a wide range of symptoms attributed to their POH and therefore felt all other patients should have the opportunity for the same. However, the difficulty with this is that large lists of symptoms for patients to select from would result in the survey for POST taking significantly longer to complete. This may result in poorer engagement of patients with the study such as patients declining to participate or failing to complete the survey fully. Reassuringly, all workshop participants felt POST in its current format was easy to complete and appropriate for patients to use both before and after surgery to assess the severity of their POH and impact on QOL, as well as the success of surgical intervention to repair their POH.

Overall, these patient workshops positively demonstrate the accuracy of and patient acceptability of the Para-oesophageal hernia SympTom tool in assessing symptom severity and impact on quality of life caused by para-oesophageal hernia. Comments about the POST tool were positive and supportive which may lead to a high number of patients agreeing to participate in the planned longitudinal cohort study. The longitudinal cohort study will further assess validity of the tool before and after POH repair, and thereby allow clinicians to standardise reporting symptoms of POH and evaluate the response to surgical intervention

**REFERENCES**

1. Patel NM, Puri A, Sounderajah V, Ferri L, Griffiths E, Low D, Maynard N, Mueller C, Pera M, van Berge Henegouwen MI, Watson DI, Zaninotto G, Hanna GB, Markar SR, Para-Oesophageal hernia Symptom Tool C. Quality of life and symptom assessment in paraesophageal hernias: a systematic literature review of reporting standards. *Dis Esophagus* 2021;**34**(7).

2. Yelisetti R, Awad A, Kaji A. Diffuse parenchymal lung disease with micro aspirations in presence of hiatal hernia. *Respir Med Case Rep* 2017;**22**: 212-214.

**Table S1: Summary of thematic findings on ‘accuracy of the POST tool in terms of the symptoms included in the tool in assessing patients with POH,’**

| **Accurate**   - ‘Ongoing chest pain and difficulties swallowing’ - ‘experienced food sticking in the chest, heart-racing and breathing difficulties’   *-the above comments by participating patients suggest the POST tool accurately captures the symptoms caused by POH* |
| --- |
| **Inaccurate**   - **‘**Didn’t have any of the symptoms mentioned’ - **‘**initially had heart investigations’ - ‘recurrent chest infections and colds for six months’ - ‘food intolerance and bowel issues’ - ‘left-sided chest pain only’ - ‘crushing chest pain’ - ‘frequent “chest colds”’   *-the above comments demonstrate that some of the initial symptoms patients suffered that led to a diagnosis of POH are not consistent with those in the POST tool* |

*Para-oesophageal hernia (POH)*

**Table S2: Summary of thematic findings on, ‘Additional symptoms reported by the participating patients that should be included in the POST tool’**

- ‘Feeling of the heart racing’
- ‘Chest and shoulder pain worse after physical exertion’
- ‘Early feeling of fullness after surgery’
- ‘Bloating’
- ‘Gassiness’
- ‘Reflux’
- ‘Chest pain and pneumonia were the most worrying’
- ‘Fatigue’
- ‘Chest and shoulder pain after re-do surgery for POH’
- ‘Chest pain not related to meals’
- ‘Shortness of breath on exertion, heart racing after meals, nausea, tiredness, low mood and depression’
- ‘Excruciating chest and back pain’
- ‘Right shoulder pain’
- ‘Shortness of breath at all times’

*-the following symptoms were felt by patients to be important in the assessment of POH and therefore should be included in POST*

*Para-oesophageal hernia (POH)*

**Table S3: Summary of thematic findings on, ‘usability of the POST tool’**

| **Comments in favour of the POST tool in its current format**   - ‘The tool is easy to complete in its current form’- agreed upon by all participants of the workshop - ‘I feel the tool is usable in its current state for patients both before and after surgery’- agreed upon by all participants of the workshop - ‘The tool would be easy to complete electronically’ - ‘My symptoms have not got better despite re-do surgery and therefore I would like to keep the same symptoms on the tool for both before and after surgery’ |
| --- |
| **Comments on how the POST tool can be improved**   - ‘A scale of 1-10 to gauge severity of symptoms would be better than 0-5 as it would be more accurate’ - ‘Numbers are subjective to the individual e.g. a 5/5 is not the same for everyone’ - ‘There should be different options for patients to complete the tool, both electronically and in paper format’ - ‘Should use as many different forms of media as possible for patients to use the tool’ - ‘I am not sure how using the tool post-op would help’ - ‘I would include all the symptoms excluded in the survey completed by the experts (Delphi) but that may not be feasible’ |

**Table S4: Summary of comments from workshop held in Barcelona, Spain**

| **Patient number** | **Comments** | **Reflux** | **Identified with symptoms in tool?** | **Electronic tool acceptable?** |
| --- | --- | --- | --- | --- |
| **1** | *20 months post-op - Toupet fundoplication*   - Primary symptoms: nocturnal dyspnoea with associated cough. | n.d. | No | No |
| **2** | *18 months post-op – Collis-Nissen operation*   - Primary symptoms: Abdominal pain not related to meals (1-2 times per month). - Post POH repair symptoms: sensation of bloating in the abdomen and rectal flatus after meals. | n.d. | No | Yes |
| **3** | *60 months post-op - Collis-Nissen operation*   - Primary symptoms: shortness of breath after meals and an early feeling of fullness after eating. | n.d. | Yes | No |
| **4** | *36 months post-op Collis-Nissen operation*   - Primary symptoms: shortness of breath on exertion. - Post POH repair symptoms: difficulty getting solid foods down (more than liquids). | n.d | Yes | No |
| **5** | *24 months post-op - Toupet fundoplication*   - Primary symptoms: difficulty getting solids foods down (and in some lesser degree liquids), and an early feeling of fullness after eating. - Post POH repair symptoms: early feeling of fullness after eating, diarrhoea - Additional symptoms reported: sensation of bloating in the abdomen after meals and bringing up undigested food. | n.d | Yes | No |
| **6** | *24 months post-op - Nissen fundoplication*   - Primary symptoms: early feeling of fullness after eating, and abdominal pain after meals. - Post POH repair symptoms: bringing up undigested food and difficulty getting solid foods down. | Yes | Yes | Yes |
| **7** | *14 months post-op - Toupet fundoplication*   - Primary symptoms: difficulty getting liquids down (not solid foods), and less frequently an early feeling of fullness after eating and ringing up undigested food. - Post POH repair symptoms: early feeling of fullness after eating and rectal flatus. | Yes | Yes | Yes |
| **8** | *36 months post-op Collis-Nissen operation*   - Primary symptoms: difficulty getting liquids down and chest pain after meals, with post prandial dyspnoea and an early feeling of fullness after eating, bringing up undigested food (more liquids than solids), voice changes and cough during day and night. - Post POH repair symptoms: rectal flatus | Yes | Yes | Yes |
| **9** | *48 months post-op - Nissen fundoplication*   - Primary symptoms: difficulty getting solid foods and liquids down, and an early felling of fullness after eating. - Post POH repair symptoms: early feeling of fullness after eating and rectal flatus. experiences difficulty getting specific solid foods down (such as walnuts), accompanied by chest pain. | n.d | Yes | Yes |
