# Supplementary material for: Development of the ParaOesophageal hernia SympTom (POST) tool
Source: Br J Surg. 2022 May 31;109(8):727–32. doi: 10.1093/bjs/znac139 (PMC10364681; doi:10.1093/bjs/znac139)
Supplement: znac139_Supplementary_Data [file znac139_supplementary_data.zip › Figure S1.docx]

Figure S1: Routinely used pre-operative investigations by participants of the scoping survey.

OGD/EGD, Oesophago-gastroduodenoscopy/Esophago-gastroduodenoscopy; CT scan, Computerised tomography Scan
