# Supplementary material for: Development of the ParaOesophageal hernia SympTom (POST) tool
Source: Br J Surg. 2022 May 31;109(8):727–32. doi: 10.1093/bjs/znac139 (PMC10364681; doi:10.1093/bjs/znac139)
Supplement: znac139_Supplementary_Data [file znac139_supplementary_data.zip › Supplementary tables.docx]

**SUPPLEMENTARY TABLE 1: Demographics of study participants**

|  |  | **Scoping Round** | **Round 1** | **Round 2** |
| --- | --- | --- | --- | --- |
|  | Total Number of Participants | 25 | 26 | 24 |
|  | Surgeon | 24 | 25 | 23 |
|  | Gastroenterologist | 1 | 1 | 1 |
| Location | North America | 13 | 9 | 11 |
|  | Europe | 8 | 6 | 9 |
|  | Australasia | 3 | 2 | 3 |
|  | Africa | 1 | 1 | 1 |
| Number of POH repairs/year | 0-10 | 4 | 4 | 4 |
| (self-reported) | 11-20 | 3 | 3 | 2 |
|  | 21-30 | 4 | 4 | 2 |
|  | 30-40 | 4 | 3 | 4 |
|  | 41-50 | 2 | 2 | 2 |
|  | 50+ | 8 | 9 | 8 |
| Gender | Female | 4 | 4 | 3 |
|  | Male | 21 | 22 | 21 |

*Para-oesophageal hernia (POH)*

**SUPPLEMENTARY TABLE 2: Summary of thematic findings on ‘accuracy of the POST tool in terms of the symptoms included in the tool in assessing patients with POH,’**

| **Accurate**   - ‘Ongoing chest pain and difficulties swallowing’ - ‘experienced food sticking in the chest, heart-racing and breathing difficulties’   *-the above comments by participating patients suggest the POST tool accurately captures the symptoms caused by POH* |
| --- |
| **Inaccurate**   - **‘**Didn’t have any of the symptoms mentioned’ - **‘**initially had heart investigations’ - ‘recurrent chest infections and colds for six months’ - ‘food intolerance and bowel issues’ - ‘left-sided chest pain only’ - ‘crushing chest pain’ - ‘frequent “chest colds”’   *-the above comments demonstrate that some of the initial symptoms patients suffered that led to a diagnosis of POH are not consistent with those in the POST tool* |

*Para-oesophageal hernia (POH)*

**SUPPLEMENTARY TABLE 3: Summary of thematic findings on, ‘Additional symptoms reported by the participating patients that should be included in the POST tool’**

- ‘Feeling of the heart racing’
- ‘Chest and shoulder pain worse after physical exertion’
- ‘Early feeling of fullness after surgery’
- ‘Bloating’
- ‘Gassiness’
- ‘Reflux’
- ‘Chest pain and pneumonia were the most worrying’
- ‘Fatigue’
- ‘Chest and shoulder pain after re-do surgery for POH’
- ‘Chest pain not related to meals’
- ‘Shortness of breath on exertion, heart racing after meals, nausea, tiredness, low mood and depression’
- ‘Excruciating chest and back pain’
- ‘Right shoulder pain’
- ‘Shortness of breath at all times’

*-the following symptoms were felt by patients to be important in the assessment of POH and therefore should be included in POST*

*Para-oesophageal hernia (POH)*

**SUPPLEMENTARY TABLE 4: Summary of thematic findings on, ‘usability of the POST tool’**

| **Comments in favour of the POST tool in its current format**   - ‘The tool is easy to complete in its current form’- agreed upon by all participants of the workshop - ‘I feel the tool is usable in its current state for patients both before and after surgery’- agreed upon by all participants of the workshop - ‘The tool would be easy to complete electronically’ - ‘My symptoms have not got better despite re-do surgery and therefore I would like to keep the same symptoms on the tool for both before and after surgery’ |
| --- |
| **Comments on how the POST tool can be improved**   - ‘A scale of 1-10 to gauge severity of symptoms would be better than 0-5 as it would be more accurate’ - ‘Numbers are subjective to the individual e.g. a 5/5 is not the same for everyone’ - ‘There should be different options for patients to complete the tool, both electronically and in paper format’ - ‘Should use as many different forms of media as possible for patients to use the tool’ - ‘I am not sure how using the tool post-op would help’ - ‘I would include all the symptoms excluded in the survey completed by the experts (Delphi) but that may not be feasible’ |

**SUPPLEMENTARY TABLE 5: Summary of comments from workshop held in Barcelona, Spain**

| **Patient number** | **Comments** | **Reflux** | **Identified with symptoms in tool?** | **Electronic tool acceptable?** |
| --- | --- | --- | --- | --- |
| **1** | *20 months post-op - Toupet fundoplication*   - Primary symptoms: nocturnal dyspnoea with associated cough. | n.d. | No | No |
| **2** | *18 months post-op – Collis-Nissen operation*   - Primary symptoms: Abdominal pain not related to meals (1-2 times per month). - Post POH repair symptoms: sensation of bloating in the abdomen and rectal flatus after meals. | n.d. | No | Yes |
| **3** | *60 months post-op - Collis-Nissen operation*   - Primary symptoms: shortness of breath after meals and an early feeling of fullness after eating. | n.d. | Yes | No |
| **4** | *36 months post-op Collis-Nissen operation*   - Primary symptoms: shortness of breath on exertion. - Post POH repair symptoms: difficulty getting solid foods down (more than liquids). | n.d | Yes | No |
| **5** | *24 months post-op - Toupet fundoplication*   - Primary symptoms: difficulty getting solids foods down (and in some lesser degree liquids), and an early feeling of fullness after eating. - Post POH repair symptoms: early feeling of fullness after eating, diarrhoea - Additional symptoms reported: sensation of bloating in the abdomen after meals and bringing up undigested food. | n.d | Yes | No |
| **6** | *24 months post-op - Nissen fundoplication*   - Primary symptoms: early feeling of fullness after eating, and abdominal pain after meals. - Post POH repair symptoms: bringing up undigested food and difficulty getting solid foods down. | Yes | Yes | Yes |
| **7** | *14 months post-op - Toupet fundoplication*   - Primary symptoms: difficulty getting liquids down (not solid foods), and less frequently an early feeling of fullness after eating and ringing up undigested food. - Post POH repair symptoms: early feeling of fullness after eating and rectal flatus. | Yes | Yes | Yes |
| **8** | *36 months post-op Collis-Nissen operation*   - Primary symptoms: difficulty getting liquids down and chest pain after meals, with post prandial dyspnoea and an early feeling of fullness after eating, bringing up undigested food (more liquids than solids), voice changes and cough during day and night. - Post POH repair symptoms: rectal flatus | Yes | Yes | Yes |
| **9** | *48 months post-op - Nissen fundoplication*   - Primary symptoms: difficulty getting solid foods and liquids down, and an early felling of fullness after eating. - Post POH repair symptoms: early feeling of fullness after eating and rectal flatus. experiences difficulty getting specific solid foods down (such as walnuts), accompanied by chest pain. | n.d | Yes | Yes |
